# Current study of pathogenetic mechanisms and therapeutics of chronic atrophic gastritis: a comprehensive review

**DOI:** 10.3389/fcell.2024.1513426

**Published:** 2024-12-10

**Authors:** Weihong Kuang, Jialin Xu, Fenting Xu, Weizhen Huang, Muhammad Majid, Hui Shi, Xia Yuan, Yongdui Ruan, Xianjing Hu

**Affiliations:** ^1^ Guangdong Provincial Key Laboratory of Research and Development of Natural Drugs, School of Pharmacy, Guangdong Medical University, Dongguan, China; ^2^ Dongguan Key Laboratory of Traditional Chinese Medicines for Prevention and Treatment of Digestive Diseases, Guangdong Medical University, Dongguan, China; ^3^ Dongguan Key Laboratory of Chronic Inflammatory Diseases, The First Dongguan Affiliated Hospital, Guangdong Medical University, Dongguan, China; ^4^ Cancer Center, The First Huizhou Affiliated Hospital, Guangdong Medical University, Huizhou, China; ^5^ Department of Acupuncture, The First Dongguan Affiliated Hospital, Guangdong Medical University, Dongguan, China

**Keywords:** chronic atrophic gastritis, pathogenetic mechanisms, gastric precancerous lesions, biomarkers, therapeutic targets

## Abstract

Chronic atrophic gastritis (CAG) is a prevalent digestive system disease characterized by atrophy of the gastric mucosa and the disappearance of inherent gastric glands. According to the theory of Correa’s cascade, CAG is an important pathological stage in the transformation from normal condition to gastric carcinoma. In recent years, the global incidence of CAG has been increasing due to pathogenic factors, including *Helicobacter pylori* infection, bile reflux, and the consumption of processed meats. In this review, we comprehensively described the etiology and clinical diagnosis of CAG. We focused on elucidating the regulatory mechanisms and promising therapeutic targets in CAG, with the expectation of providing insights and theoretical support for future research on CAG.

## 1 Introduction

Chronic atrophic gastritis (CAG) is one of the most prevalent gastrointestinal diseases, characterized by gastric mucosa atrophy, reduction of glandular structures, and mucosal nodules. CAG is one of the precancerous lesions of gastric cancer. Patients diagnosed with CAG usually suffer from nausea, loss of appetite, indigestion, vomiting, upper abdominal pain, and loss of weight (De Vries et al., 2008; Miceli et al., 2012).

Gastric cancer (GC) continues to rank as the fifth most common cancer and the fourth highest cause of cancer-related mortality globally. It is estimated that the global burden of GC will increase by 62% by 2040 (Thrift et al., 2023). The multifactorial progression of GC development, known as Correa’s cascade, comprises multiple sequential events (Piazuelo et al., 2021). Chronic inflammation constitutes the initial step in this cascade, leading to persistent irritation of the gastric glands and parietal cell atrophy (Kuligowski et al., 2016). With the aggravation of gastric mucosal inflammation, the second process is gastric precancerous lesions including chronic non-atrophic gastritis, intestinal metaplasia, and dysplasia (Si et al., 2024). The third phase of Correa’s cascade is gastric tumorigenesis. Therefore, timely and effective treatment of CAG is of great significance for the prevention of GC.

In this review, we will broadly delineate the etiology and contemporary diagnostic approaches of CAG. The research focus on targets and signaling pathways regulating CAG, which is beneficial for enhance our understanding of its pathogenesis and develop novel therapeutic interventions for CAG patients.

## 2 The etiology of CAG

The etiology of CAG is complicated and is associated with a variety of factors, which may act synergistically. The main causes for the development of CAG including *Helicobacter pylori* infection, bile reflux, N-nitroso compounds exposures, high salt intake, alcohol consumption, chronic persistent stress and immunologic factor.

### 2.1 *Helicobacter pylori* infection

CAG has several known inducers with the most common being *Helicobacter pylori* (Hp) infection. A recent cluster-randomized controlled trial shows that mass screening for Hp and its eradication can reduce the incidence of GC, thereby providing further evidence for the role of Hp in the pathogenesis of CAG and GC (Pan et al., 2024).

Hp-induced CAG is mainly mediated by the virulence factors VacA and CagA (Keates et al., 1997). The pathological roles of CagA involve regulating multiple signaling pathways in the host cells. Type IV secretory system (T4SS) is used to inject CagA into gastric epithelial cells, leading to the activation of downstream signaling pathways. Following injection, CagA undergoes tyrosine phosphorylation, contributing to DNA damage and the trigger of the Wnt/β-catenin pathway (Bauer et al., 2020). CagA has been observed to interact with E-cadherin, resulting in the translocation of β-catenin to the nucleus and the activation of Wnt target genes (Kurashima et al., 2008; Murata-Kamiya et al., 2007). Studies have observed that CagA enhances the degradation of the p53 tumor suppressor and reverses the p53-regulated apoptotic response (Buti et al., 2011). Moreover, the inactivation of the p53 tumor suppressor induces chromosomal or genomic instability and DNA double-strand breaks (Hanahan and Weinberg, 2011; Zamperone et al., 2019). VacA is the second most frequently studied Hp virulence factor. VacA can induce multiple pathological activities, including the inducement of vacuolation, release of cytochrome C from mitochondria, and trigger of inflammatory response (Camilo et al., 2017). In addition, VacA can also inhibit the activation and proliferation of immune cells (Cover and Blanke, 2005; Foegeding et al., 2016). Therefore, Hp infection plays a vital role in accelerating the pathology of CAG.

### 2.2 Bile reflux

Recently, accumulating evidence has demonstrated that bile reflux is also a key pathogenic factor in CAG and has been identified as an independent risk factor for GC according to an observational cross-sectional study (Zhang et al., 2021). Furthermore, another clinical study has provided histological evidence suggesting a close correlation between bile reflux and intestinal metaplasia (Dixon et al., 2002).

Bile reflux is characterized by the backflow of duodenal contents, such as bile and duodenal juice, into the gut. In physiological conditions, the majority of bile acid is secreted into the intestine lumen and circulates back to the liver. The gut microbiome facilitates the transformation of primary bile acids to secondary bile acids by dehydroxylation. Bile reflux into the stomach causes gastric mucosal dysfunction by inducing cell apoptosis, enhancing cell permeability with intercellular junctions damaged, which contributes to the aggravation of gastric inflammation (Goldman et al., 2010; Shi et al., 2016).

Farnesoid X receptor (FXR) is a key bile-acid binding receptor that plays an essential role in bile acid homeostasis. Research has demonstrated that FXR expression is significantly increased in intestinal metaplasia tissues (Qu and Shi, 2022). Numerous studies have shown that the NF-κB signaling pathway is associated with the regulation of FXR expression (Zhou et al., 2018). Recent studies further elucidated that bile reflux can alter the abundance and composition of gastric microbiota because of alkaline bile. Based on the 16S rRNA analysis, the pathogenic bacteria *Comamonas*, *Halomonas,* and *Shewanella* are found to be enriched in patients with bile reflux, which may contribute to the aggravation of CAG (Huang et al., 2022).

### 2.3 N-nitroso compounds exposure

Evidence has demonstrated that exposure to N-nitroso compounds (NOCs) is an essential factor contributing to CAG and the progression of GC. The common NOCs, including nitrosamines and nitrosamides, exhibit mutagenic, genotoxic, and carcinogenic effects in both experimental animals and humans (Kobayashi, 2018). People can be exposed to NOCs via the consumption of processed meats and cigarette smoking, both of which contribute to the endogenous formation of NOCs (Kuhnle et al., 2007; Tricker et al., 1991). It is widely recognized that NOCs in common dietary sources can be formed by bacterial nitrite reductases and increased pH in the stomach (Kobayashi, 2018).

It is widely proved that the underlying mechanisms by which exposure to NOCs promotes CAG and GC involve multiple targets and signaling pathways, including gene mutation and pro-inflammatory signaling pathways. Recent studies have shown that 1-methyl-3-nitro-1-nitrosoguanidine (MNNG) and N-methyl-N-nitrosourea (MNU) can facilitate N6-methyladenosine (m^6^A) modifications, thereby promoting the progression of GC (Song et al., 2024; Wang Q. et al., 2024). Additionally, a study on human epithelial cells has revealed that MNNG regulates the downstream pro-angiogenic factors of NF-κB by enhancing the phosphorylation of NF-κB p65, which may contribute to the increased incidence of CAG and GC (Chen Y. et al., 2018).

### 2.4 High salt intake

According to a retrospective review, a high-salt diet is closely associated with Hp infection and the progression of CAG and GC (Song et al., 2017). Significant advancements have been made in elucidating the mechanisms underlying the induction of CAG by high salt intake. An *in vivo* study shows that a high-salt diet induces malignant cell proliferation and lipid peroxidation (Vences-Mejía et al., 2005). Another study reveals that high salt intake increases the expression levels of cyclooxygenase-2 (COX-2) and inducible nitric oxide synthase (iNOS). Therefore, research has confirmed that the COX-2 inhibitor, celecoxib, can prevent the development of GC in the rodent model (Hu et al., 2004). In addition, evidence suggests a notable increase in inflammatory cytokines, including TNF-α, IL-1β, IL-6, and IFN-γ, in mice subjected to a high-salt diet (Leung et al., 2008).

### 2.5 Other etiological factors

Alcohol consumption is an essential independent risk factor for CAG and gastric ulcers. A clinical study has confirmed that the severity of gastric atrophy is closely correlated with alcohol consumption and frequency of drinking (Ozeki et al., 2024). Accumulating evidence indicates that alcohol can induce gastric injury through multiple mechanisms, mainly including the activation of oxidative stress and local inflammatory response (Liu F. et al., 2022). Other researchers have unveiled that excessive ethanol intake can damage gastric mucosal microcirculation and disrupt cell proliferation, resulting in activation of the NF-κB signaling cascade (Yu L. et al., 2020).

Chronic persistent stress is also an important inducer of CAG. Researchers usually use a mouse model subjected to water avoidance stress to stimulate the long-term psychological stress that contributes to the development of CAG (Bradesi et al., 2005). It is widely accepted that stress events can induce gastric acid secretion. Further studies have demonstrated that stress-induced parasympathetic overactivity and an increase in vagal outflow may contribute to excessive gastric acid secretion (Guo et al., 2012). A recent study has shown that the maintenance of gastric microbiota homeostasis is also an essential factor in stress-induced CAG (Han et al., 2020).

The immunologic factor is also a causative factor of CAG. Autoimmune atrophic gastritis is an autoimmune disease, characterized by the gastric corpus-fundus mucosa damaged (Massironi et al., 2019). The presence of anti-parietal cell antibodies can contribute to autoimmune atrophic gastritis (Goldenring, 2023). It is well-established that these anti-parietal cell antibodies demonstrate complement-dependent cytotoxic activity *in vitro*. However, the pathogenesis of autoimmune atrophic gastritis is still controversial. Accumulating evidence suggests that pro-inflammatory cytokines, including TGF-β, TNF-α, and IL-6, are involved in the development of autoimmune atrophic gastritis (Cascetta et al., 2024). Similarly, Hp infection has been found to promote the progress of autoimmune atrophic gastritis. The interaction between Hp antigens and gastric H^+^/K^+^ ATPase is considered a potential pathogenic mechanism underlying this process (Massironi et al., 2019).

## 3 Clinical diagnosis of CAG

Over the past decades, there are several diagnostic methods for CAG. In this section, we provide a summary of the main clinical diagnosis methods, including endoscopy, pathological diagnosis and gastric functioning markers. We also discuss some potential biomarkers on CAG diagnosis, which may provide novel solutions to early clinical diagnosis.

### 3.1 Endoscopy and pathological diagnosis

Given that the symptoms exhibited by patients are typically subtle, early diagnosis is crucial. The CAG diagnosis mainly relies on gastroscopy and pathological analysis of biopsies. Histopathological assessment of gastric biopsies of antrum and corpus mucosa is the gold standard for the diagnosis of CAG. The typical endoscopic features of CAG include the loss of gastric folds, a pallid appearance of the gastric mucosa, and the thinning of gastric mucosa (Shah et al., 2021). The OLGA/OLGIM classifications are used for the diagnosis and classification of CAG. However, gastroscopy cannot be performed frequently in a short time. Furthermore, gastroscopy causes damage to the esophagus, leading to patient discomfort and psychological pressure (Gai et al., 2023).

Recently, deep learning and artificial intelligence have been found to assist in the diagnosis of CAG. Studies have shown that deep learning models are superior to endoscopists and exhibit higher levels of accuracy, suggesting that they have better clinical diagnostic value (Turtoi et al., 2024; Zhao et al., 2022).

### 3.2 Gastric functioning markers

Serum biomarkers are beneficial for the diagnosis of CAG, including gastrin, pepsinogen (PG I, PG II), and gastric proton pump H^+^/K^+^ ATPase (Jia et al., 2024). Gastrin expression is upregulated in many gastric carcinomas of the stomach corpus (Smith et al., 2017), including endocrine tumors (Massironi et al., 2009). A large-scale retrospective cohort study shows that gastrin measurement is essential for the risk evaluation of GC (Nagasaki et al., 2023). Some study also indicate that gastrin level is an important marker for predicting the polyp risk in autoimmune gastritis patients (Massironi et al., 2024; Massironi et al., 2023). The low PG I level or low ratio between PG I and PG II usually indicate the presence of CAG. In addition, the pepsinogen level can help distinguish CAG and autoimmune atrophic gastritis (Chapelle et al., 2023). According to a prospective case-finding study, gastric proton pump H^+^/K^+^ ATPase is an effective biomarker for the identification of CAG in high-risk patients (Lahner et al., 2020).

### 3.3 Other potential biomarkers

**FIGURE 1:**
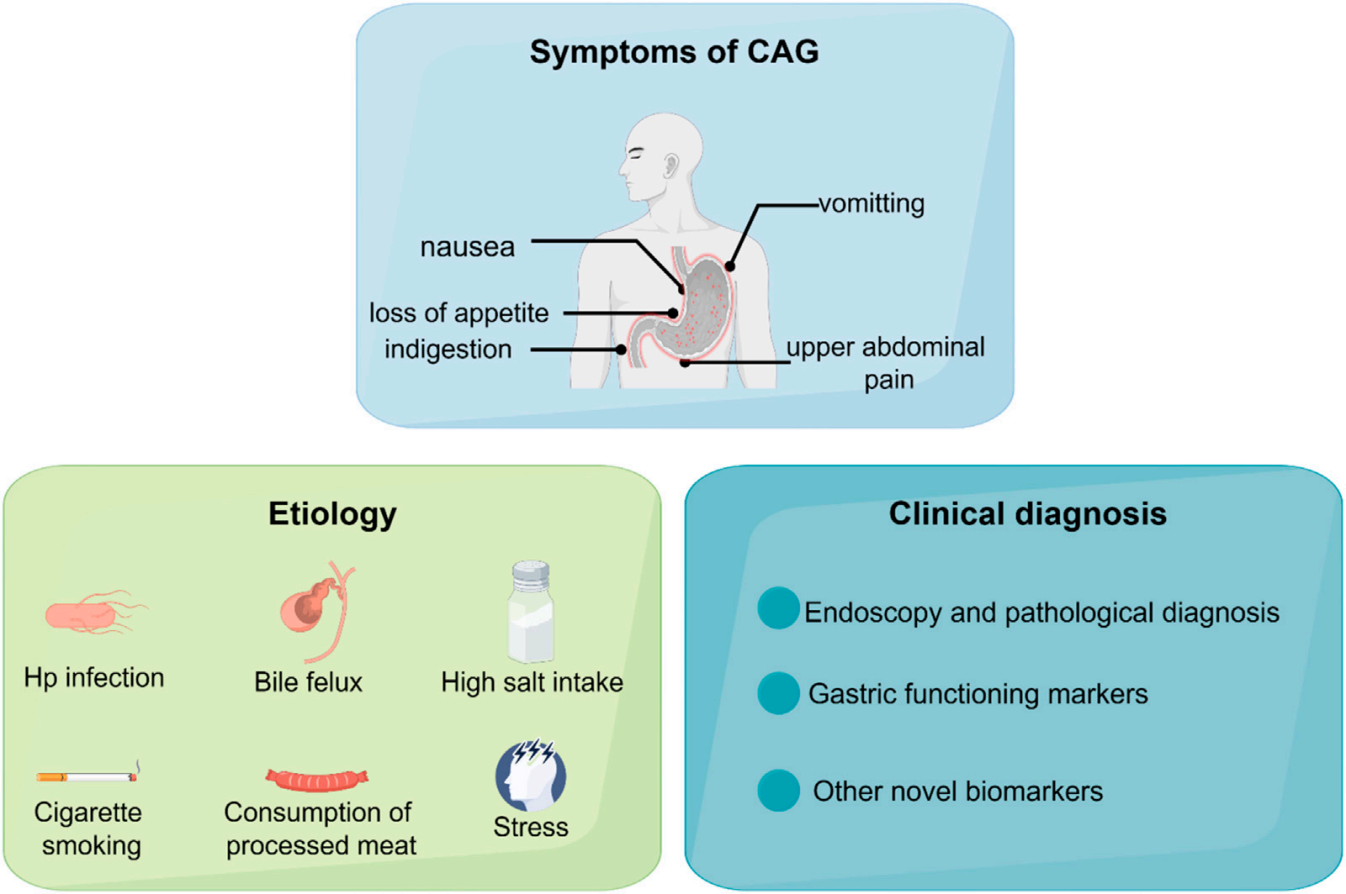
The symptoms, etiology, and clinical diagnosis of CAG.

In recent years, numerous novel noninvasive diagnostic methods have been discovered. Based on the ^1^H NMR-based metabonomics results, arginine, succinate, 3-hydroxybutyrate, valine, and α-ketoglutarate have potential as indicators of CAG risk (Cui et al., 2017). These metabolites have specificity and sensitivity, which have potential to act as early diagnosis biomarkers for CAG. In China, traditional Chinese medicine doctors usually observe the changes in patients’ tongues to diagnose diseases, because it is well-known that the changes in tongues are influenced by the physiological function of the stomach. In addition, in a study comparing gastritis patients with healthy individuals, researchers have found that *Campylobacter concisus* is detected in tongue coating, indicating tongue coating microbiota is a promising non-invasive biomarker for CAG (Cui et al., 2019). This study provides a non-invasive and individualized complement for CAG diagnosis. Numerous researches also show that gastric microbiome and serum proteins could be biomarkers for distinguishing CAG cases (Aziz et al., 2022; Dong et al., 2023) (Figure 1). Chromogranin A (CgA) is also identified as an effective biomarker to serve as indicating the histological degree of enterochromaffin-like (ECL) cell lesions, which reflect the severity of CAG. However, CgA is of limited clinical diagnosis utility because of a lack of sensitivity and specificity (Al-Toubah et al., 2019).

## 4 Programmed cell death in CAG

**FIGURE 2:**
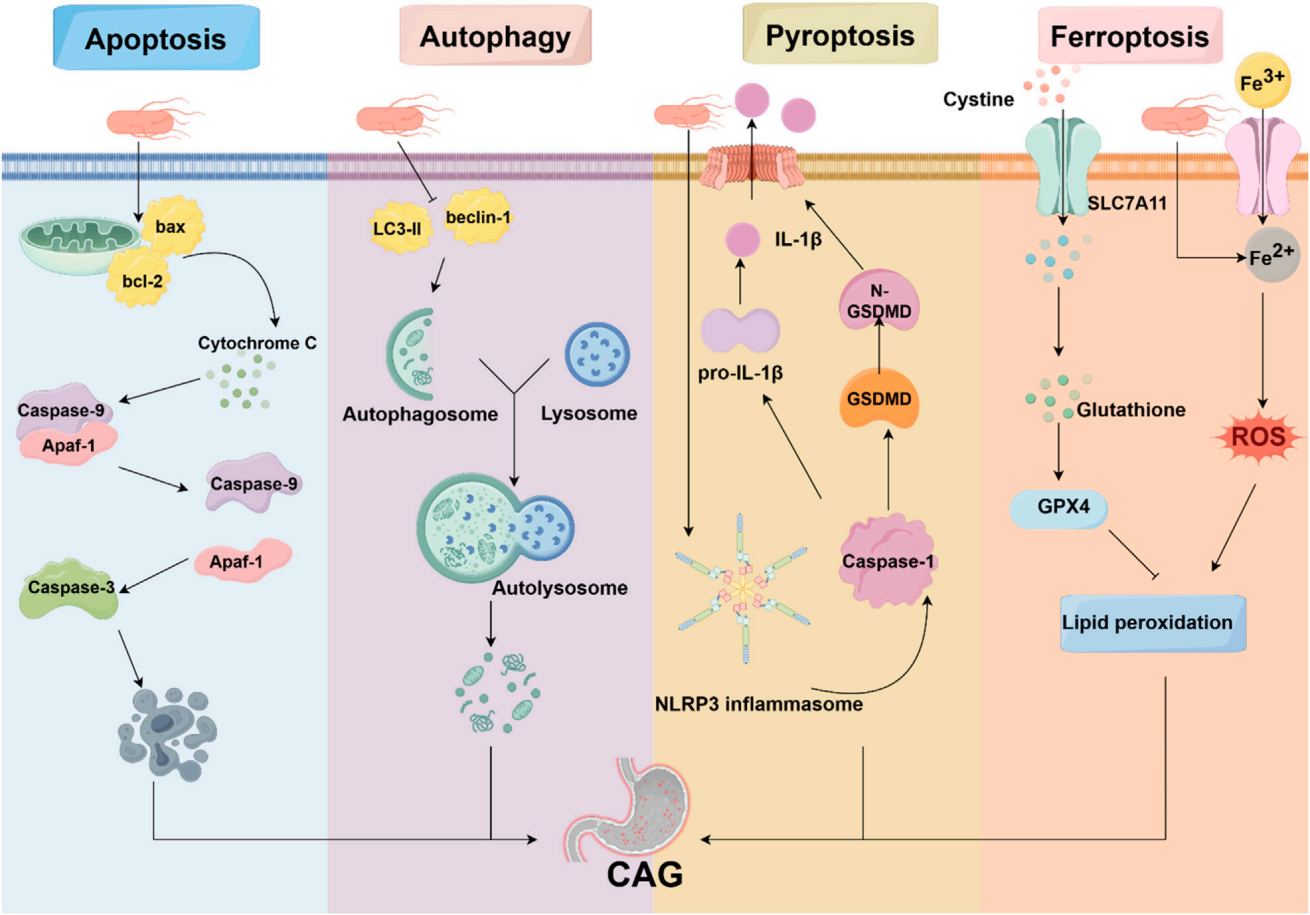
Programmed cell death in CAG. Programmed cell death is involved in the pathogenesis of CAG, including apoptosis, autophagy, pyroptosis, and ferroptosis. HP, acting as an avital pathogenic factor, can regulate programmed cell death and affect the development of CAG.

Programmed cell death is characterized by the orderly death of cells, regulated by genes for the stability of the internal environment, including apoptosis, autophagy, ferroptosis, pyroptosis, and necroptosis. It is well established that programmed cell death plays various roles in the pathogenesis of CAG (Figure 2).

### 4.1 Apoptosis

Apoptosis is widely regarded as a caspase-mediated form of programmed cell death that plays a major role in the control of cell proliferation and tissue homeostasis (Chen Q. et al., 2018). The morphological characteristics of apoptosis include chromatin condensation, cell shrinkage, and nuclear fragmentation (Lockshin and Zakeri, 2001). The mitochondria-dependent apoptosis is activated by diverse signals such as oxidative stress and DNA damage, leading to the activation of pro-apoptotic factors. The death receptor-mediated apoptosis is triggered by the binding of Fas ligand, TNF-α, or TRAIL (Wang and El-Deiry, 2003).

It is generally recognized that the pathogenesis of CAG is strongly associated with apoptosis. Immunohistochemistry results of the clinical gastric resection specimens have shown that CAG and intestinal metaplasia are related to an increase in apoptotic gastric epithelial cells (Van Grieken et al., 2003). Increasing evidence suggests that Hp induces gastric epithelial cell apoptosis (Moss et al., 1996; Yamaguchi et al., 2000). According to electron microscopic observations, the virulence factor of Hp, VacA, induces apoptosis in GES-1 cells (Yuan et al., 2021). *In vitro* studies have provided the molecular mechanisms by which HP promotes apoptosis. AGS cells depleted of tumor necrosis factor receptor-associated protein 1 (TRAP1) increases apoptosis (Teng et al., 2019). However, the effect of Hp on apoptosis is not unidirectional, suggesting that the imbalance between proliferation and apoptosis contributes to Hp-induced CAG and GC. A study shows that Hp activates anti-apoptotic pathways to inhibit gastric epithelial self-renewal, leading to enhanced Hp colonization (Mimuro et al., 2007). Taken together, apoptosis is strongly related to CAG, and the medicines with anti-apoptotic activity can be used for future anti-CAG research.

### 4.2 Autophagy

Autophagy is a process involving the formation of autophagosomes to clear and degrade pathogens, damaged organelles, and unfolded proteins (Shu et al., 2023). The process of autophagy involves four predominant steps: autophagy initiation, vesicle nucleation, autophagosome formation, and hydrolyzation (Mizushima and Levine, 2020). Recent research has shown that several key autophagy-related genes, including ATG7, ATG16L1, and ATG12, are implicated in the progression of CAG and potentially serve as biomarkers, suggesting that autophagy is strongly associated with the pathogenesis of CAG (Yamaguchi et al., 2024; 2023).

However, autophagy plays a dual role in the development of CAG. On the one hand, the inhibition of autophagy exacerbates the apoptosis of GES-1 cells and gastric mucosa damage caused by aspirin (Hernández et al., 2016). Further studies show that the activation of autophagy by regulating mTOR can enhance apoptosis and oxidative stress in GES-1 cells (Chang et al., 2017). During the Hp infection, the inhibition of autophagy leads to DNA damage in Hp-infected cells and the development of gastric tumorigenesis via Rad51 ubiquitination (Xie et al., 2020). Several antioxidant agents can upregulate autophagy and downregulate apoptosis to prevent Hp-infected gastric injury (Yang and Chien, 2009).

On the other hand, uncontrolled autophagy leads to aberrant cell proliferation, which plays a key role in the development of CAG (Lu et al., 2021). The autophagy-related gene ATG16L1 has been revealed to increase the risk of Hp-induced inflammation. In addition, VacA is found to induce autophagy and cell death in gastric epithelial cells by regulating the endoplasmic reticulum stress pathway (Zhu et al., 2017). Therefore, researchers have found that clearance of autophagosomes offers a strategy to decrease Hp colonization (Zhang et al., 2018).

### 4.3 Pyroptosis

Pyroptosis is a new form of programmed cell death characterized by the activation of caspase family proteins. This activation leads to the cleavage of gasdermin D (GSDMD), ultimately leading to cell membrane disruption (Newton et al., 2024; Yu P. et al., 2021). In the classical pathway, inflammasomes are activated by pathogenic exposures, resulting in the recruitment and activation of caspase-1. Subsequently, GSDMD is cleaved, causing plasma membrane disruption and causing cytoplasmic efflux. Simultaneously, cleaved caspase-1 also activates inflammatory factors, including IL-1β and IL-18. The non-classical pathway is activated by lipopolysaccharide (LPS). Caspase-11, caspase-4, or caspase-5 can bind to LPS and hydrolyze GSDMD, triggering membrane perforation (Zhou et al., 2023).

A recent study has shown that pyroptosis is involved in the pathogenesis of gastritis and the progression of GC. Researchers constructed a cellular CAG model using GES-1 cells, discovering alterations in their morphology and plasma membrane (Zhou Z. et al., 2024). In addition, pyroptosis is also associated with Hp infection. The virulence factors of Hp, including CagA, VacA, FlaA, and UreB, regulate the inflammasome machinery and the release of pro-inflammatory cytokines (Kumar and Dhiman, 2008).

The NOD-like receptor protein 3 inflammasome (NLRP3) inflammasome is an important executor of pyroptosis. NLRP3 can combine with ASC and NEK7 to form the NLRP3 inflammasome, which activates caspase-1 that cleaves GSDMD into N-GSDMD (Sun et al., 2024). It has been recognized that the CagA protein of Hp activates the NLRP3 inflammasome, and silencing of NLRP3 can reverse the effect of Hp on cell migration (Zhang X. et al., 2022). Moreover, Hp has also been discovered to induce IL-18 and IL-1β production through activation of NLRP3 inflammasome (Li et al., 2015).

### 4.4 Ferroptosis

Ferroptosis is a distinct iron-dependent cell death characterized by the accumulation of lipid peroxides and redox imbalance (Zhou Q. et al., 2024). The morphological features of ferroptosis include mitochondria with condensed membranes and decreased cristae (Cao and Dixon, 2016; Dixon et al., 2012). Mechanistically, intracellular iron accumulation contributes to lipid peroxidation by the Fenton reaction and the production of reactive oxygen species (ROS) (Yan et al., 2021). Additionally, glutathione (GSH)-glutathione peroxidase 4 (GPX4) antioxidant system, as the key repressor of ferroptosis, plays a vital role in protecting cells from ferroptosis. It is observed that GSH serves as the electron donor for reducing toxic phospholipid hydroperoxides to non-toxic phospholipid alcohols (Seibt et al., 2019).

According to the multi-omics integration and molecular docking results, researchers confirm that the ferroptosis-related gene YWHAE is involved in the progression of CAG and GC (Liu D. et al., 2023). In addition, a recent study reveals that Hp infection increases the sensitivity of cells to RAS-selective-lethal 3 (RSL3)-induced ferroptosis by regulating phosphorylase kinase G2 (PHKG2) (Zhu et al., 2023). Hepcidin is an iron-regulatory hormone, that can regulate the cellular iron exporter (Nemeth and Ganz, 2023). Studies have revealed that hepcidin expression is elevated in CAG gastric tissue via the IL-6/STAT3 signaling pathway (Zhao et al., 2023). Furthermore, gastric hepcidin is highly expressed during Hp infection but normalized after eradication, suggesting that hepcidin may become a future target for CAG treatment by inhibiting ferroptosis (Schwarz et al., 2012).

## 5 Molecular pathways and key targets in the pathogenesis of CAG

**FIGURE 3:**
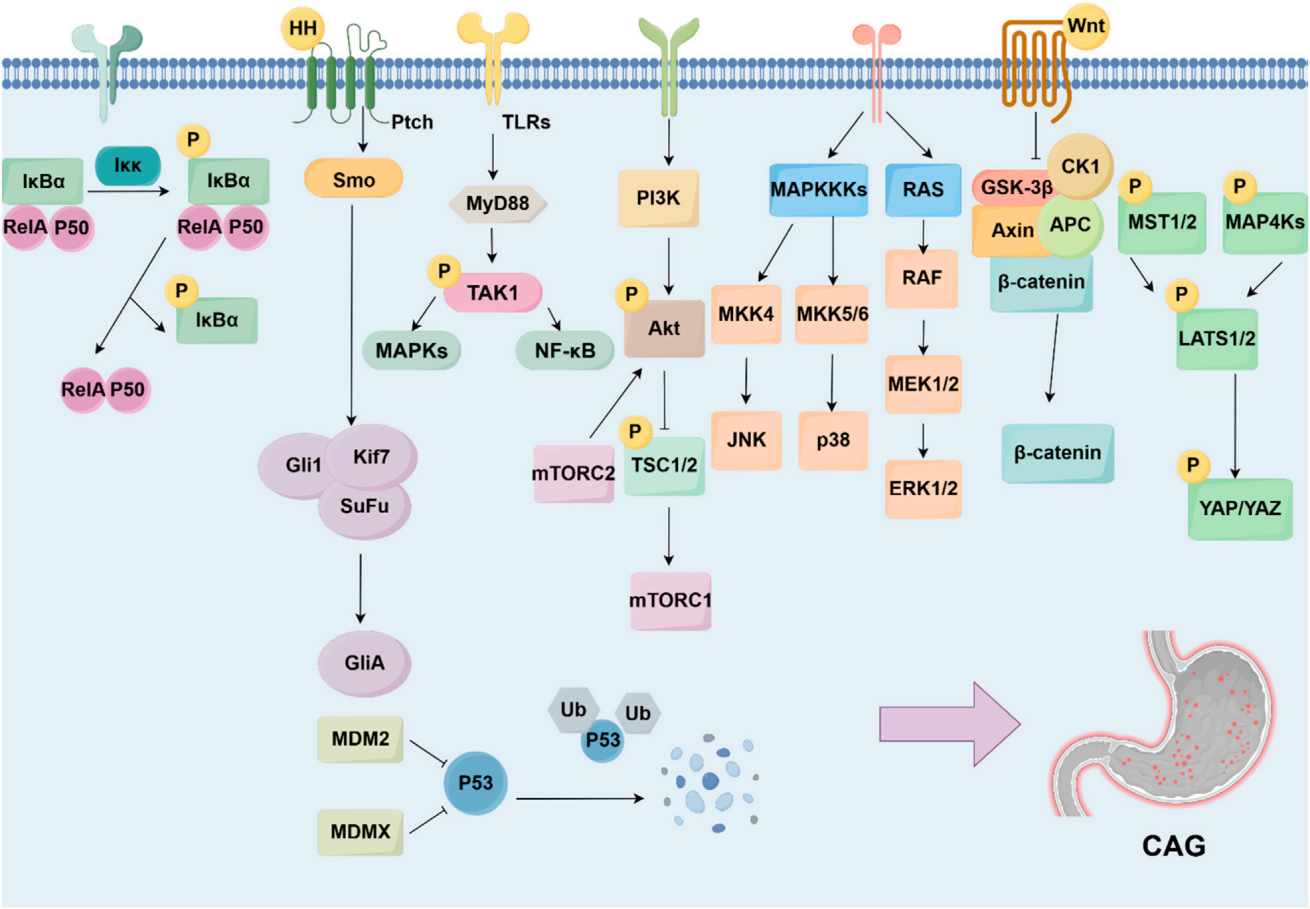
Signaling pathways and targets regulating CAG development. The development of CAG is achieved by numerous signaling pathways and targets, including NF-κB pathway, Hedgehog pathway, TLRs pathway, PI3K/Akt pathway, MAPK pathway, Wnt/β-catenin pathway, p53 pathway, and Hippo pathway.

Numbers of signaling pathways and the key targets were reported to be related with CAG development, including the NF-κB, Hedgehog pathway, TLRs pathway, PI3K/Akt pathway, MAPK pathway, Wnt/β-catenin pathway, p53 pathway, and Hippo pathway (Figure 3).

### 5.1 NF-κB signaling pathway

NF-κB family includes five members, p65 (RelA), RelB, c-Rel, p105/p50, and p100/p52. The activation of NF-κB starts with the phosphorylation of IκB, induced by IKKs, leading to the release of NF-κB. The released NF-κB translocates to the nucleus and drives the transcription of targeted genes (Yu H. et al., 2020). It is widely recognized that NF-κB signaling pathway dysfunction has been associated with various human diseases, including inflammation, malignancies, and autoimmune disorders (Guo et al., 2024). Existing studies show that targeting the NF-κB signaling pathway can alleviate gastric damage and suppress inflammation, which is essential for the pathogenesis of CAG (Kim et al., 2021). For instance, the NF-κB inhibitor pyrrolidine dithiocarbamate (PDTC) has been found to inhibit excessive cell proliferation and reverse CAG in mice (Jiang et al., 2021).

Studies have demonstrated that activated NF-κB upregulates chemokines or adhesion molecules, thereby promoting inflammation in Hp-associated gastritis (Isomoto et al., 2000; Maubach et al., 2022). Similarly, conditional deletion of IκB promotes Hp-induced cell apoptosis and the development of dysplasia (Shibata et al., 2010). Meanwhile, Hp also induces the NF-κB signaling pathway by its effector ADP-heptose, leading to the establishment of a persistent effect. A diverse range of molecules regulate the activity of NF-κB in Hp-infected gastritis. For instance, the p53 upregulation modulator of apoptosis (PUMA), as a pro-apoptotic protein, has been discovered that NF-κB binds to PUMA’s promoter to contribute to the pathogenesis of Hp-infected gastritis (Dang et al., 2020).

### 5.2 Hedgehog signaling pathway

The Hedgehog signaling pathway is a pathway regulating embryonic development and tissue homeostasis. It includes three ligands, including Sonic hedgehog (Shh), Indian hedgehog (Ihh), and Desert hedgehog (Dhh), as well as three Gli proteins, Gli1, Gli2, and Gli3 (Jiang, 2022). It has become clear that gastric epithelial cells can secrete Shh, which contributes to the renewal of epithelial cells in response to gastric injury (Konstantinou et al., 2016). Therefore, accumulating evidence shows that the loss of the Hedgehog signaling pathway is an important indicator of Hp-associated CAG progressing to GC (Shiotani et al., 2005). The malfunction of the Hedgehog signaling pathway influences the progression of CAG, including the decrease in gastric acid secretion and the loss of parietal cells (Wessler et al., 2017).

Hp is reported to directly control the expression level of the Shh signal. A study shows that Hp-induced IL-1β inhibits the expression level of Shh, contributing to gastric atrophy (Waghray et al., 2010). However, in certain conditions, Hp-induced caudal-type homeobox 2 (Cdx2) can bind to the promoter of the Shh gene, leading to the downregulation of Shh (Shiotani et al., 2006). It has also been revealed that the Hedgehog signaling pathway acts as a macrophage chemoattractant and affects the immune response (Schumacher et al., 2012).

### 5.3 Toll-like receptors signaling pathway

Toll-like receptors (TLRs) can identify pathogen-associated molecular patterns (PAMPs), which mediate the immune response and activate intracellular signaling pathways, especially the MyD88-mediated pathway (Barton and Medzhitov, 2003). It is widely recognized that the TLR signaling pathway plays an essential role in immune response regulation and several inflammatory diseases.

Many studies on CAG have reported that the TLR signaling pathway is closely correlated with the progression of gastritis (Table 1). TLR4 gene polymorphisms have been found to mediate the inflammatory response in gastritis (Zhang Z. et al., 2024). Moreover, studies have shown that TLR9 promotes gastric tumorigenesis and facilitates Hp-infected gastritis (Tang et al., 2022). The most widely studied is the myeloid differentiation factor-88 (MyD88)-mediated signaling pathway. TLRs regulate inflammation through MyD88. It has been reported that TLRs interact with Hp by binding to MyD88, leading to the activation of the NF-κB signaling pathway and the release of inflammatory factors. Numerous studies show that the TLRs/MyD88 signaling pathway can increase Hp chemotaxis and promote cell migration through a diverse range of inflammatory and oncogenic pathways (Liu M. et al., 2023).

**TABLE 1:** Roles of Toll-like receptors in CAG.

Types of Toll-like receptors	Mechanism of action	References
TLR4	TLR4 recognizes LPS of HP to trigger inflammatory response and generates pro-inflammatory cytokines. TLR4/MyD88/NF-κB signaling cascade involves in inflammatory response	Schmidinger et al. (2022), Zhang Z. et al. (2024)
TLR8	TLR8 involves in HP-induced Type I interferon and phosphorylation of interferon regulatory factor 7(IRF7)	Lee et al. (2022)
TLR2	TLR2 has an immuno-regulatory role during HP infection	Nemati et al. (2017)
TLR10	TLR10 mediates NF-κB activation, recognizes LPS and involves in the innate immune response	Nagashima et al. (2015)
TLR5	The virulence factor of HP, CagL, mediates NF-κB activation in TLR5-dependent manner	Pachathundikandi et al. (2019)
TLR9	TLR9 mediates HP-induced myeloid-derived suppressor cells polarization. But other studies show that TLR9 inhibits the HP-infected inflammation	Ding et al. (2022), Otani et al. (2012)
TLR6	TLR6 increases the expression levels of IL-1β and IL-8, recruiting neutrophils and reducing the HP colonization	Zhang X. et al. (2024)
TLR1	TLR1 has an influence on the generation of IFN-γ in NK cells and T cells	Yang et al. (2013)

### 5.4 PI3K/Akt signaling pathway

The PI3K/Akt signaling pathway is a major signaling pathway that controls cell survival and metabolism (He et al., 2021). The PI3K/Akt signaling pathway belongs to the family of serine/threonine protein kinases and starts with the activation of receptor tyrosine kinases (RTKs) by growth factors. It is involved in the modulation of numerous downstream targets, which include NF-κB, mTOR, and MDM2 activation (Lei et al., 2022). Research shows that the PI3K/Akt signaling pathway affects neutrophils and lymphocytes, resulting in the promotion of inflammation.

Based on immunohistochemistry analysis of biopsy tissue, researchers discovered that the levels of p-PI3K and p-Akt in Hp-positive patients are higher than those in Hp-negative patients (Xie and Liu, 2018). A study demonstrates that Hp infection regulates eukaryotic protein translation by activating the PI3K/Akt signaling pathway, which can be reversed by the PI3K inhibitor, LY294002 (Sokolova et al., 2014). Hp also regulates downstream targets to affect the progression of CAG. Researchers have found that Hp regulates FoxO1/3a in gastric epithelial cells, which regulates the host immune response and cell apoptosis (Tabassam et al., 2012).

### 5.5 MAPK signaling pathway

The mitogen-activated protein kinases (MAPK) are a group of serine/threonine kinases and play a pivotal role in cell proliferation and differentiation (Bahar et al., 2023). The MAPK family consists of three major kinases: c-JUN N-terminal kinases (JNKs), extracellular signal-regulated kinases (ERKs), and p38 (Johnson and Lapadat, 2002). There is increasing evidence that the MAPK signaling pathway is an essential regulator of inflammatory diseases, including pancreatitis, acute colitis, and gastritis (Hong et al., 2024; Junyuan et al., 2018; Zhang J. et al., 2022). In addition, the activation of the MAPK signaling pathway is associated with the pathology of gastric mucosal injury (Arab et al., 2019; Fu et al., 2018). Immunostaining results reveal that Hp-induced changes in the gastric mucosa are associated with the activation of the MAPK signaling pathway (Kacar et al., 2007).

During Hp infection, the Hp structural compound CagL interacts with integrin α5β1 on gastric epithelial cells, leading to the activation of the MAPK signaling pathway (Gorrell et al., 2013). Moreover, it has been reported that oligomerization domain 1 (NOD1) is required for MAPK activation during Hp infection (Allison et al., 2009).

MAPK activation leads to numerous pathological processes. Hp induces overexpression of MMP-3 and MMP-9 via the MAPK signaling pathway, leading to the disturbance of host cellular signaling and cell adhesion (Karayiannis et al., 2023). Another study also shows that Hp increases the secretion of gastrin by MEK1, ERK2, and c-RAF in the MAPK signaling pathway (Gunawardhana et al., 2018).

### 5.6 Wnt/β-catenin signaling pathway

The Wnt/β-catenin signaling pathway is an essential pathway that plays roles in regulating cell proliferation, cell metabolism, cancer metastasis, and cancer immunity (Liu J. et al., 2022). The Wnt family includes Wnt3a, Wnt1, Wnt5a, and so on (Yu F. et al., 2021). When extracellular Wnt ligands bind to membrane receptors, the levels of β-catenin accumulate and are transferred to the nucleus, eventually becoming involved in the expression of downstream targeted genes, including c-myc and cyclin D1 (Reyes et al., 2020). It has been discovered that the Wnt/β-catenin signaling pathway functions as a molecular target for pathogenic bacteria, including Hp (Silva-García et al., 2019). In addition, it has been elucidated that the Wnt/β-catenin signaling pathway is involved in the progression of CAG, as it is recognized as a key regulator of EMT. Studies show that MNNG stimulation combined with Hp infection induces EMT by enhanced expression and activation of Wnt2 and β-catenin (Lin et al., 2019).

Aquaporin 5 (AQP5) is a member of the AQP family, which plays an essential role in modulating water transport (Tan et al., 2020). Hp infection has been reported to increase the expression level of AQP5 in gastric epithelial cells (Li et al., 2021). In addition, bioinformatics results suggest that achaete-scute complex-like 1 (ASCL1) binds to AQP5 (Wang et al., 2017). Therefore, researchers have proven that Hp infection activates the Wnt/β-catenin signaling pathway via upregulating ASCL1/AQP5 to induce CAG (Zuo et al., 2022).

### 5.7 p53 signaling pathway

TP53 is an important tumor suppressor gene that plays a vital role in regulating cell proliferation and apoptosis, and it has been considered a focus of cancer research (Kastenhuber and Lowe, 2017; Levine, 2020). Mutations in TP53 are strongly associated with the process of tumorigenesis. The p53 protein is promoted for degradation through ubiquitination by the negative regulators MDM2 and MDMX in the normal cellular environment (Bieging et al., 2014). When exposed to internal and external stresses, intracellular p53 protein is accumulated because of the cessation of p53 ubiquitination (Wang et al., 2023). Eventually, activated p53 binds to DNA and modulates gene transcription. In addition, numerous studies have shown that p53 also regulates other signaling pathways, including autophagy, ferroptosis, cellular senescence, and so on (Spike and Wahl, 2011; White, 2016).

The p53 signaling pathway is associated with Hp infection. It has been reported that Hp induces gastritis in p53-knockout mice compared to wild-type mice, suggesting that p53 plays an important role in Hp-associated CAG (Nagata et al., 2004). Similarly, the severity of CAG is closely related to p53 mutations (Taguchi et al., 2006). The underlying potential mechanisms have been discovered and reported. Hp has been shown to activate Akt1, resulting in the degradation of p53 (Wei et al., 2010). In addition, research shows that p53 and CXCL12 promote cellular senescence, leading to gastric mucosal atrophy (Cai et al., 2021).

### 5.8 Hippo signaling pathway

The Hippo signaling pathway plays a vital role in modulating cell proliferation, cell differentiation, and cell survival (Fu et al., 2022). The key components of the Hippo signaling pathway include mammalian STE20-like kinase1/2 (MST1/2), protein Salvador homolog 1 (SAV1), large tumor suppressor kinase 1/2 (LATS1/2), Yes-associated protein (YAP), and WW-domain-containing transcription regulator (TAZ) (Liu Y. et al., 2023). Dysregulation of the Hippo signaling pathway is associated with a variety of diseases.

Some findings underscore the fact that dysregulation of the Hippo signaling pathway is involved in the progression of Hp-induced CAG and GC. Researchers have shown that the expression of YAP in gastric tissue of Hp-positive patients is higher than that in Hp-negative patients (Li et al., 2018). It has been reported that Hp-related virulence factors, in combination with the Hippo signaling pathway, promote the processes of inflammation and carcinogenesis (Messina et al., 2023). Studies indicate that CagA promotes the delocalization of ZO-1 and ZO-2, thereby increasing the expression level of YAP. In turn, activation of YAP/TAZ also contributes to Hp infection and promotes the process of EMT (Tiffon et al., 2020). Additionally, Hp-induced YAP has been found to promote IL-1β expression, thereby mediating gastric carcinogenesis (Wu et al., 2019). However, a study has revealed that the Hippo signaling pathway protects gastric cells from Hp-induced EMT and metaplasia. The results show that LATS2 restricts Hp-induced EMT and metaplasia phenotype, thereby contributing to the control of the progression of CAG and GC (Molina-Castro et al., 2020). In conclusion, the Hippo signaling pathway exhibits dual effects in Hp-mediated CAG.

## 6 Novel mechanisms regulating CAG development

**FIGURE 4:**
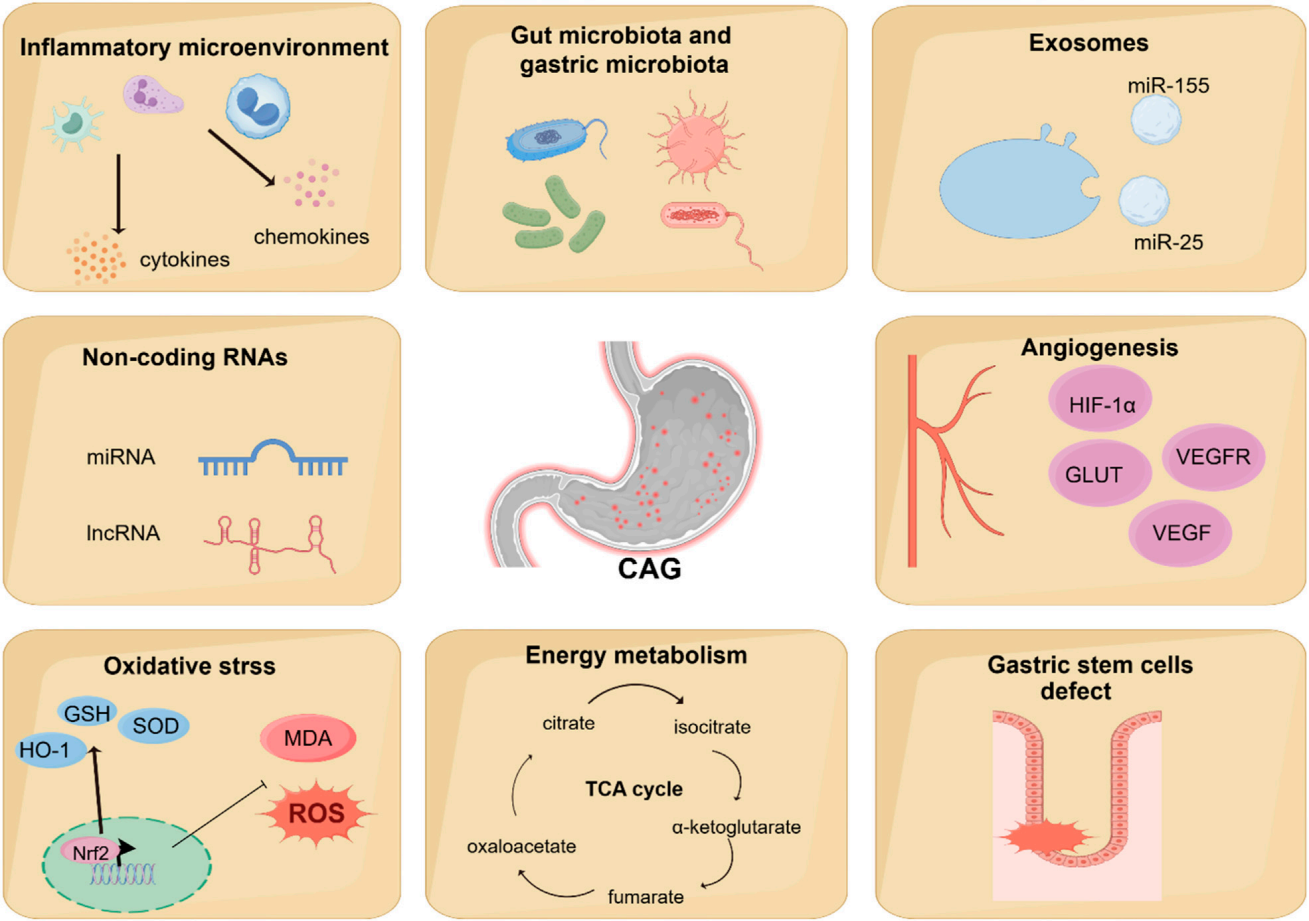
Novel mechanisms regulating CAG development. Recent studies have found that other novel mechanisms are associated with the regulation of CAG development, including angiogenesis, energy metabolism, gut microbiota and gastric microbiota, inflammatory microenvironment, oxidative stress, gastric stem cells defect, non-coding RNAs, and exosomes.

Numerous studies have shown the classic signaling pathways are associated with the development of CAG, but some novel mechanisms involved in the CAG has been discovered, which may provide a more comprehensive understanding of the pathogenesis of CAG. Recent studies have shown that angiogenesis, energy metabolism, gut microbiota and gastric microbiota, inflammatory microenvironment, oxidative stress, gastric stem cells defect, non-coding RNAs, exosomes are associated with CAG (Figure 4).

### 6.1 Angiogenesis

Angiogenesis is a physiological process involving the development of new blood vessels from existing ones. It is well-established that angiogenesis plays a vital role in maintaining homeostasis. However, because of the overexpression of pro-angiogenic factors and the inactivation of anti-angiogenic factors, angiogenesis dysregulation occurs in a variety of immune diseases. Furthermore, studies have shown that solid tumor cells, in response to a hypoxic microenvironment, secrete pro-angiogenic factors to promote the formation of new blood vessels. The vascular endothelial growth factor (VEGF) is the most important in the angiogenic system, as it mediates both vascular permeability and angiogenesis. Studies have shown that the imbalance of angiogenic factors and angiogenesis may play an important physiological role in the development of CAG (Kawano and Tsuji, 2000).

HIF-1α participates in regulating the relative expression of VEGF and facilitating the process of angiogenesis in hypoxic conditions (Kitajima and Miyazaki, 2013). Research has shown that the expression levels of HIF-1α, VEGF, VEGFR2, Pacilin, and SRC are upregulated in the CAG model group compared to control groups (Liu et al., 2024; Wen J.-X. et al., 2021).

Glucose transporters (GLUTs) are a kind of carrier of glucose for transporting and widely exist in cells (Ni et al., 2020). Numerous studies have shown that GLUTs are closely related to tumor angiogenesis (Airley and Mobasheri, 2007). Overexpression of GLUTs is frequently observed in tumors, with Glut1, 3, 4, 6, 10, and 12 being expressed in gastric carcinoma (Pujol-Gimenez et al., 2015; Schlößer et al., 2017). A previous study has shown that the expression levels of Glut4 and Glut1 significantly increase in precancerous lesions of gastric cancer (Zeng et al., 2022).

Recent research has demonstrated that the Notch signaling pathway also plays a pivotal role in angiogenesis. In mammals, there are three Notch receptors: Notch2, Notch3, and Notch4. In humans and mice, five Notch ligands have been identified, including the Serrate-like ligands Jagged1 (JAG1) and JAG2, as well as the delta-like ligands DLL1, DLL3, and DLL4 (Zhou et al., 2022). It is reported that overexpression of DLL4 is associated with poor prognosis in GC patients (Du et al., 2014). Furthermore, the upregulation of DLL4 has been confirmed to promote angiogenesis in the gastric tissue of gastric precancerous lesion groups (Gao et al., 2022).

### 6.2 Energy metabolism

In eukaryotes, a small amount of ATP is produced in the cytoplasm by glycolysis, but most of the ATP is produced in the mitochondria via oxidative phosphorylation. Mitochondrial function also plays a vital role in regulating lipid biosynthetic pathways and maintaining the levels of numerous metabolites. Research has elucidated that alterations in mitochondrial energy metabolism may represent an important mechanism underlying CAG, particularly in association with deficiencies in the respiratory complex I of mitochondria (Gruno et al., 2008). Similarly, according to the metabolomics-based network pharmacology, it has been reported that there exists a link between mitochondrial energy metabolism dysfunction and CAG (Cui et al., 2017; Liu et al., 2019).

More and more evidence has shown that Hp infection alters metabolites in host lesions, including the tricarboxylic acid (TCA) cycle, choline pathway, and urea cycle (Nishiumi et al., 2017). A urinary metabolomics and network pharmacology study revealed that CAG model rats show a reduction in α-ketoglutarate level, which may be attributed to dysfunction of the TCA cycle and disrupted energy metabolism (Liu et al., 2019). Further research has also shown that Hp infection damages mitochondrial activity, affecting a wide range of metabolites and metabolic pathways (Machado et al., 2013). Additionally, another study shows that Hp infection may result in alterations in energy metabolism accompanied by an increase in both glycolysis and oxidative phosphorylation (Zhu et al., 2024).

Three branched-chain amino acids (BCAAs), namely, leucine, isoleucine, and valine, play crucial roles in numerous physiological functions (Sivanand and Vander Heiden, 2020). Growing evidence indicates that BCAAs are implicated in a wide range of chronic human diseases, such as type 2 diabetes and cardiovascular diseases (White and Newgard, 2019). Meanwhile, two aromatic amino acids (AAAs), tyrosine and phenylalanine, are also essential amino acids that have been linked to the progression of chronic disease. An increase in BCAAs is related to the progression of CAG. According to the 1H NMR metabolomics results, it is evident that the levels of BCAAs and AAAs are significantly increased in the autoimmune CAG group (Xu et al., 2022).

### 6.3 Gut microbiota and gastric microbiota

Alterations in gut microbiota composition have been associated with various digestive disorders, including irritable bowel syndrome (IBS) and colorectal cancer (Thumann et al., 2019). Accumulating research has shown that changes in the diversity and abundance of gut microbiota play a crucial role during the progression from CAG to GC (Zhao and Yu, 2024). Based on 16s rRNA gene sequencing results, the progression from CAG to GC is characterized by a decrease in beneficial bacteria, such as *Akkermansia*, and enrichment of pathogens, such as *Escherichia_Shigella* (Yu C. et al., 2020).

It is generally recognized that microorganisms are unlikely to colonize the stomach because of its acidic conditions. However, recent research has found that Hp infection can lead to increased pH levels, which may allow other gastric microbes to colonize the stomach (Stewart et al., 2020). Meanwhile, an imbalance in the gastric microbiome is tightly associated with gastric diseases, including gastritis, gastric ulcer, and GC (Sohn et al., 2017; Yu et al., 2017). *In vivo* studies have also shown that gastric microbes contribute to the progression of GC after Hp eradication (Sung et al., 2020). Specifically, a bacterium called *P. melaninogenica* has been observed to be obviously increased in the gut of patients with bile acid reflux gastritis and GC. This bacterium produces LPS and interacts with TDCA, eventually promoting gastric carcinogenesis (Wang et al., 2022). In addition, a cross-sectional, monocentric study of patients with CAG revealed a higher colonization of *Streptococcus*, which is linked to a higher risk of GC (Conti et al., 2021). Evidence suggests that *Streptococcus anginosus* can cause acute inflammatory responses, parietal cell atrophy, and metaplasia (Fu et al., 2024).

Therefore, probiotic intervention is considered as an adjuvant therapy for CAG treatment. A open randomized clinical trial indicates that probiotics significantly improve the Hp eradication effect and limit the progression of CAG (Du et al., 2012). In GES-1 cell experiment, researchers have confirmed that *L. acidophilus* and *L. bulgaricus* obviously inhibit Hp adherence to GES-1 cells and regulate TLR4/IκBα/NF-κB signaling pathway (Song et al., 2019). Moreover, probiotics have been observed to regulate the gastric microbiota to alleviate Hp-induced gastric inflammation (He et al., 2022). A study shows that *Lacticaseibacillus paracasei* positively modulate the gastric microbiome and alleviate CAG in Hp-infected mice (Yu J. et al., 2024). Another experiment also confirms similar view. *Lacticaseibacillus paracasei* strain LPG-9 is identified as an anti-inflammatory, anti-Hp and gastroprotective probiotic, which is beneficial for CAG treatment (Xu et al., 2023).

### 6.4 Inflammatory microenvironment

It is well established that the inflammatory microenvironment is modulated by the development and progression of CAG. Inflammatory factors play a vital role in coordinating the inflammatory microenvironment (Table 2). Studies have confirmed that increased level of IL-1β is one of the feature of CAG, contributing to the regulation of mucosa atrophy (Ding et al., 2021). IL-13 acts directly on gastric epithelium cells, leading to metaplastic changes in epithelial cells during the progression from CAG to GC (Noto et al., 2022). Elevated levels of IL-17A induce apoptosis in gastric parietal cells and are related to the disease severity of CAG (Bockerstett et al., 2018). Interferon-γ (IFN-γ), a cytokine secreted by T helper cell 1(Th1 cells), contributes to the release of pro-inflammatory factors. It has been reported that IFN-γ treatment can cause mucous neck cell hyperplasia (Kang et al., 2005).

**TABLE 2:** Cytokines and chemokines in CAG.

Type	Name	Implicated in CAG	References
Cytokine	IL-6	HP infection can induce IL-6 production	Yu B. et al. (2024)
Cytokine	IFN-γ	IFN-γ is directly involved in the death of gastric epithelial cells	Tu et al. (2012)
Cytokine	IL-13	IL-13 directly influences gastric epithelial cells	Noto et al. (2022)
Cytokine	IL-27	Reducing the severity of inflammation mice with gastritis	Bockerstett et al. (2018)
Cytokine	IL-21	IL-21 is elevated in the inflammatory gastric mucosa	Nishiura et al. (2013)
Cytokine	IL-17	IL-17A neutralizing antibody reduced parietal cell atrophy	Bockerstett et al. (2018)
Chemokine	CXCL8	Recruitment of neutrophils	Allen (2007)
Chemokine	CXCL10	Recruitment of Th1 cells. Contributes to the induction suitable Th1 cell-mediated immune response against HP	Minaga et al. (2018)
Chemokine	CXCL12	Promotion of the inflammation-related cells	Capitani et al. (2019)
Chemokine	CXCL13	Playing a significant role in the formation of gastric lymphoid follicles through binding to its receptor CXCR5	Finch et al. (2013)
Chemokine	CXCL16	Induction of anti-HP immune responses	Lv et al. (2019)
Chemokine	CCL5	Recruitment of eosinophils and memory T cells	Kikuchi et al. (2000)
Chemokine	CCL17	Recruitment of Treg cells	Mizukami et al. (2008)
Chemokine	CCL20	Induction of anti-HP immune responses. Maintenance of a chronic inflammation	Tsai and Hsu (2017)
Chemokine	CCL22	Recruitment of Th2 cells and Treg cells	Al-Balushi et al. (2016), Liu et al. (2015)
Chemokine	CCL25	Recruitment macrophages	Laing and Secombes (2004)
Chemokine	CCL28	Recruitment of IgA-producing cells	Hansson et al. (2008)

Peripheral blood monocytes are differentiated into M0 macrophages triggered by macrophage colony-stimulating factor (M-CSF). M0 macrophages can be polarized into M1 macrophages by LPS stimulation, or into M2 macrophages by M2 stimuli (Bosco, 2019). M1 macrophages are capable of producing pro-inflammatory factors including IL-1β, IL-6, IFN-γ, and TNF-α, thereby facilitating the pro-inflammatory response (Murray et al., 2014). In contrast, M2 macrophages can release anti-inflammatory factors such as TGF-β, IL-10, and CD206, contributing to the anti-inflammatory response (Ivashkiv, 2013). Macrophages exert significant effects on HP-induced gastritis. It has been reported that HP promotes M1 polarization by upregulating MAPK, NF-κB, and Notch signaling pathways (Tang et al., 2021; Wen J. et al., 2021). In addition, analysis of gastric specimens from humans and mice with Hp infection shows that the infiltration of M1 macrophages is increased compared to that of M2 macrophages, leading to the production of pro-inflammatory substances (Wei et al., 2024). Studies have revealed that quercetin reverses Hp-infected damage to gastric epithelial cells by regulating M1 macrophage polarization via the SP1/LCN2 axis (Wang Z. et al., 2024).

### 6.5 Oxidative stress

During the progression of CAG, excessive amounts of ROS and reactive nitrogen species (RNS) are released in the inflammatory tissues of the stomach (Mousavi et al., 2020). The accumulation of ROS is well-recognized for its pivotal role in the pathogenesis of chronic inflammatory disorders. Researchers use the biomarkers of oxidative stress to investigate the effects of Hp eradication. The results show that the expression of iNOS and the production of nitric oxide (NO) are decreased after Hp eradication treatment (Pignatelli et al., 1998). As another evidence of oxidative stress related to CAG, researchers have observed that the level of malondialdehyde (MDA) is higher in the gastric tissue of Hp-infected patients (Wang et al., 2018).

The virulence factors of Hp contribute significantly to the oxidative stress observed in Hp-infected CAG. Studies have shown that higher hydrogen peroxide levels are measured with CagA-positive strains. Furthermore, VacA is capable of the activation of NF-κB signal and the generation of ROS. In addition, the virulence factors of Hp contribute to the accumulation of TNF-α and IL-8, which are important oxidative stress markers.

Hp-induced oxidative stress may contribute to DNA damage (Han et al., 2022). Studies have reported an increase in the level of γH2AX during Hp infection, serving as a marker for double-strand DNA breaks (Pignatelli et al., 2001). Further study shows that Hp-infected AGS cells exhibit a higher expression level of apurinic/apyrimidinic endonuclease 1 (APE1), potentially making it more difficult to repair DNA mutations (Manoel-Caetano et al., 2019).

### 6.6 Gastric stem cells defect

Gastric stem cells have the potential for self-renewal and differentiation, which contribute to maintaining the homeostasis of the stomach (Hata et al., 2018). In the corpus gland, gastric stem cells are marked by SOX2, LRIG1, and TFF2 (Liu Meng et al., 2023). In the antral gland, gastric stem cells are marked by LGR5, AXIN2, and AQP5 (Xiao and Zhou, 2020). Recent data supports the notion that gastric stem cell defect is an essential pathogenic factor of CAG and GC. By analyzing MNNG-induced CAG model mice and gastric organoids, a recent study shows that gastric stem cell defect is an essential CAG pathogenic factor associated with the decreasing of the EGF signaling pathway (Li et al., 2024). Additionally, another recent study has proved that gastric isthmus stem cells, marked by Mist1, promote gastric injury and inflammation by Wnt5a (Nienhüser et al., 2021).

In addition, accumulating evidence has proved that there is an interaction between gastric stem cells and Hp in the pathogenesis of CAG. Studies have shown that Hp targets Lrig1+ gastric stem cells, which contributes to increased inflammation and malignant proliferation in gastric (Wroblewski et al., 2019). Hp promotes the activation of the R-spondin 3 (Rspo3) signal, which drives the expansion of gastric stem cells. It is well recognized that Lgr4 is a target gene of Rspo3. Therefore, studies have revealed that gastric stem cells promote Hp-induced gastritis via the Rspo3-Lgr4 axis (Wizenty et al., 2022).

### 6.7 Non-coding RNAs

It is well known that non-coding RNAs (ncRNAs) are major RNA transcripts characterized by the ability to regulate gene expression by epigenetic mechanisms (Chen et al., 2022). The most studied types of ncRNAs include microRNAs (miRNAs), circularRNAs (circRNAs), and long-non-coding RNAs (lncRNAs). An increasing number of studies have identified that ncRNAs play vital roles in many diseases.

MiRNAs are a family of endogenous non-coding RNAs that modulate a diverse range of biological processes. It has become clear that miRNA expression is dysregulated in many human diseases, including cancers of the upper gastrointestinal tract (Peng and Croce, 2016). A single-center cross-sectional study in CAG patients has identified that the expression of miR-146a and miR-370 is substantially downregulated in these patients, suggesting that the dysregulation of miR-146a and miR-370 may be an indicator of early gastric neoplasia (Pita et al., 2021). Another study found that miR-196a-5p is related to the progression of CAG to GC and targets ACER2 for inducing malignant cell proliferation (Zheng et al., 2023). In addition, miRNA dysregulation has been involved in Hp-mediated gastritis and gastric carcinoma. MiRNAs have been reported to be mediators of host immune response by regulating TLRs signaling pathway and NF-κB signaling pathway (Săsăran et al., 2021). Moreover, compelling evidence has demonstrated that miRNAs and Hp regulate cell cycle, cell invasion, and metastasis (Noto and Peek, 2012).

LncRNAs are defined as RNAs over 200 nucleotides long that are not translated into proteins. It is widely believed that lncRNAs play an essential role in physiological and pathological processes and are tightly related to regulating cell cycle, cell apoptosis, and cell proliferation. Studies have suggested that lncRNAs polymorphisms are associated with CAG and GC, indicating that lncRNAs might be biomarkers for the diagnosis of GC (Liu X.-Y. et al., 2022; Petkevicius et al., 2020). Additionally, METTL3-regulated lncRNA SNHG7 has been reported to drive MNNG-induced gastric precancerous lesions and the EMT process (Jian et al., 2024).

### 6.8 Exosomes

Exosomes are a subset of extracellular vesicles that are secreted by eukaryotic cells and play a key role in intercellular communication (Dai et al., 2020). Exosomes participate in a diverse range of physiological and pathological processes, such as cell differentiation, cell migration, and cancer development. In addition, increasing studies have proven that exosomes can be used as biomarkers for disease diagnosis (Tan et al., 2024).

Many studies have shown that exosomes play an important role in CAG. Evidence has reported that Hp infection can induce the upregulation of miR-155 in exosomes secreted by macrophages, leading to the accumulation of pro-inflammatory cytokines such as IL-6, TNF-α, and IL-23 (Yao et al., 2015). Similarly, another research found that miR-25 levels are elevated in exosomes derived from GES-1 cells after Hp infection, resulting in upregulating NF-κB signaling pathway and the release of pro-inflammatory factors (Li et al., 2019).

## 7 Recommended treatment and management options for CAG

### 7.1 Lifestyle intervention

The development of CAG is tightly associated with living habits. Smoking, a high-salt diet, and the consumption of processed meat are linked to an increased prevalence of CAG. Dietary intervention is important for the prevention and treatment of CAG. For instance, studies have found that green tea extract can inhibit the process of CAG and gastric tumorigenesis in mouse models (Jeong et al., 2016). A study also shows that daily intake of sulforaphane-rich broccoli sprouts can prevent Hp and high-salt-induced gastric atrophy (Yanaka et al., 2009). In addition, a buckwheat diet is found to alleviate CAG in C57BL/6 mice, and an oat β-glucans diet can reverse CAG in human patients (Gudej et al., 2021; Li et al., 2020). Early symptoms of CAG can be reversed by improvements in lifestyle interventions and the side effects of non-pharmacological interventions are minimal. However, lifestyle interventions will not inhibit the development of disease in the later stages of CAG. Therefore, drug intervention is still essential.

### 7.2 Gastric acid secretion regulation medicines

The decreased gastric acid secretion is a major characteristic of CAG. Excessive gastric acid secretion causes histamine to bind to histamine 2 receptors, leading to the release of gastric acid into the gastric lumen (Engevik et al., 2020). H2 receptor antagonists and proton pump inhibitors are the most used medications to regulate gastric acid secretion. The representative drugs are ranitidine, nizatidine, omeprazole, and rabeprazole. In a clinical trial, a combination of ranitidine and omeprazole was found to improve the regulation of gastric acid secretion (Abdul-Hussein et al., 2015).

However, compelling evidence has demonstrated that long-term use of proton pump inhibitors is associated with complications such as GC, gastric neuroendocrine tumors, chronic kidney diseases, and heart failure (Abrahami et al., 2022; Cavalcoli et al., 2015; Hart et al., 2019; Ohori et al., 2023). A study has also revealed that exposure to proton pump inhibitors accelerates the senescence of endothelial cells by reducing telomere length (Yepuri et al., 2016). Given the severity of side effects, it is essential to reduce the unnecessary use of proton pump inhibitors (Farrell et al., 2022; Lee and McDonald, 2020).

### 7.3 Hp eradication medicines

Hp is a common worldwide pathogen that causes CAG, which can progress to severe gastrointestinal diseases. It is now well known that gastric carcinoma has become a preventable disease through the eradication of Hp. Conventional treatment regimens for Hp eradication include triple therapy and quadruple therapy (Chey et al., 2017). A standard triple therapy consists of a proton pump inhibitor and two antibiotics, such as clarithromycin and amoxicillin. The quadruple therapy is composed of a proton pump inhibitor, bismuth, and two antibiotics (Liu Y. et al., 2018).

However, the eradication rate of Hp is decreasing because of antibiotic resistance (Larsson and Flach, 2022). The underlying mechanisms driving this resistance include impaired regulation of drug uptake and chromosomally encoded mutations (Tshibangu-Kabamba and Yamaoka, 2021). In addition, the recurrence rate of Hp has shown an increasing trend over the past 10 years after eradication (Zhao et al., 2021). Studies have also revealed that Hp eradication therapy may lead to an increase in the level of pathogenic antibiotic-resistant bacteria in the gut (Hsu et al., 2019; Liou et al., 2020).

### 7.4 Gastric mucosal protective agents

The protection of the structure and physiological functions of the gastric mucosa plays an important role in CAG treatment. The gastric mucosal integrity is maintained by a variety of mechanisms, including pre-epithelial factors, the generation of PGs, surface epithelial cells connected with mucus, trefoil peptides, and so on (Laine et al., 2008). The most commonly used gastric mucosal protective agents include sucralfate, bismuth potassium citrate, and teprenone, which are reported to enhance mucus synthesis and secretion.

### 7.5 Traditional Chinese medicines treatment

Traditional Chinese medicines (TCM) play a vital role in the treatment of CAG and have gotten more attention for clinical applications. It is well known that TCM includes bioactive ingredients, proprietary Chinese medicines, and classical prescription medicines. Studies have shown great potential for TCM in the field of CAG treatment due to its multi-target regulatory abilities and low toxicity.

In a randomized double-blind clinical trial, treatment with Weierkang Pills was found to regulate the levels of trefoil peptides 3 (TFF3) and endothelial growth factor in biopsy specimens (Chen et al., 2023). In addition, numerous randomized clinical trials have confirmed that Moluodan protects gastric mucosa and reverses CAG without drug-induced side effects (Zou et al., 2024). Numerous classical prescriptions have also been confirmed to alleviate CAG, including Sijunzi Decoction, Huangqijianzhong Decoction, and Huazhuojiedu Decoction (Hao et al., 2022; Liu W. Z. et al., 2018; Zhu et al., 2024).

Many bioactive ingredients have also been proven to treat CAG. Astragaloside IV, a saponin derived from *Astragalus membranaceous* Bge, has been found to modulate the Ambra1/Beclin1 complex and inhibit autophagy, which contributes to the treatment of MNNG-induced gastric precancerous lesions (Cai et al., 2018). Kaempferol, a natural ingredient in Chaishaoliujun Decoction, can significantly decrease the expression of Shh, Ptch1, and Gli at both protein and mRNA levels, suggesting that it can inactivate the Hedgehog signaling pathway (Tu et al., 2022). Curcumin has also been found to inhibit the expression of TLRs and MyD88 in the Hp-infected mouse model (Santos et al., 2015).

TCM treatment is suitable for long-term use in CAG due to its lower toxicity and minimal side effects. However, there are some limitations in the development of TCM as a treatment for CAG. Toxicological and pharmacokinetic studies should be conducted to validate the safety of TCM. Furthermore, novel experimental methods should be developed to investigate the mechanisms of action of TCM. In addition, double-blind clinical trials are a crucial process for the future development of TCM.

## 8 Conclusion

Chronic atrophic gastritis (CAG) is a slowly progressing and intricate inflammatory disorder of the digestive system that imposes considerable economic and social consequences on individuals and society. Correa’s cascade indicates that CAG is a pivotal stage in the transition from gastritis to gastric cancer. *Helicobacter pylori* (Hp) infection significantly contributes to the onset of chronic non-atrophic gastritis, which evolves into CAG over decades, finally resulting in intestinal metaplasia and invasive cancer. In addition to its association with stomach cancer progression, CAG has been connected to compromised bone health, depression, and iron deficiency anemia (Aasarød et al., 2016; Nahon et al., 2003; Zhao et al., 2018). The prevention, early detection, and treatment of CAG necessitate considerable focus due to its extensive health ramifications. This review methodically analyzes the pathogenetic mechanisms of CAG, emphasizing the significance of programmed cell death and critical signaling pathways, including NF-κB, Hedgehog, PI3K/Akt, MAPKs, Wnt/β-catenin, p53, and Hippo. Furthermore, novel pathways including angiogenesis, energy metabolism, gut and stomach microbiome, inflammatory microenvironments, oxidative stress, gastric stem cell abnormalities, non-coding RNAs, and exosomes are examined, providing significant insights into prospective treatment targets. Although these pathways provide significant potential for enhancing CAG management, several, including non-coding RNAs and exosomes, are still in the preliminary phases of investigation. Obstacles, including the translation of molecular discoveries into clinical application and the management of patient variability, must be surmounted to optimize their potential. Future research must concentrate on discovering novel biomarkers for early detection, formulating targeted therapeutics to prevent the progression of CAG to gastric cancer, and utilizing modern technical approaches, such as proteomics and metabolomics, to elucidate regulatory mechanisms. By prioritizing these issues, researchers can facilitate the development of safer and more effective therapy strategies, hence enhancing outcomes for patients with CAG.
